# Safety and feasibility of laparoscopic resection of abdominal neuroblastoma without image-defined risk factors: a single-center experience

**DOI:** 10.1186/s12957-023-02997-9

**Published:** 2023-03-28

**Authors:** Saishuo Chang, Yu Lin, Shen Yang, Wei Yang, Haiyan Cheng, Xiaofeng Chang, Zhiyun Zhu, Jun Feng, Jianyu Han, Qinghua Ren, Huanmin Wang, Hong Qin

**Affiliations:** 1 Department of Oncology Surgery, Beijing Children’s Hospital, Capital Medical University, National Center for Children’s Health, Beijing, 100045 China; 2 MOE Key Laboratory of Major Diseases in Children, Beijing Children’s Hospital, Capital Medical University, National Center for Children’s Health, Beijing, 100045 China

**Keywords:** Neuroblastoma, Laparoscopic surgery, Open surgery, IDRF, INRG

## Abstract

**Objective:**

To explore the criteria, safety and efficacy of laparoscopic surgery in pediatric neuroblastoma (NB).

**Methods:**

A retrospective study of 87 patients with NB without image-defined risk factors (IDRFs) between December 2016 and January 2021 at Beijing Children’s Hospital was conducted. Patients were divided into two groups according to the surgical procedure.

**Results:**

Between the 87 patients, there were 54 (62.07%) cases in the open surgery group and 33 (37.93%) cases in the laparoscopic surgery group. There were no significant differences between the two groups regarding demographic characteristics, genomic and biological features, operating time or postoperative complications. However, in terms of intraoperative bleeding (*p* = 0.013) and the time to start postoperative feeding after surgery (*p* = 0.002), the laparoscopic group was obviously better than the open group. Furthermore, there was no significant difference in the prognosis between the two groups, and no recurrence or death was observed.

**Conclusion:**

For children with localized NB who have no IDRFs, laparoscopic surgery could be performed safely and effectively. Surgeons who are skilled in this can help children reduce surgical injuries, speed up postoperative recovery, and obtain the same prognosis as open surgery.

**Supplementary Information:**

The online version contains supplementary material available at 10.1186/s12957-023-02997-9.

## Introduction

Neuroblastoma (NB) is the most common extracranial solid tumor in children [1]. The treatment of NB usually consists of surgery, chemotherapy, radiation, hematopoietic stem-cell transplant (HSCT), and immunotherapy. Among them, surgery plays an important role in the local control of NB, especially in the treatment of nonhigh-risk NB [2]. Compared to laparotomy, laparoscopic resection has some advantages of less invasiveness and rapid recovery; however, the laparoscopic treatment of NB in children is still in the exploratory stage because the role of laparoscopy for NB is still controversial [3, 4]. Many studies regarding the criteria of laparoscopic surgery for NB focus on tumor size and vascular invasion [3, 5], but tumor size is not the major limiting factor for laparoscopic surgery. Image-defined risk factors (IDRFs) were proposed by the International Neuroblastoma Risk Group (INRG) to predict the surgical risks of localized NB in 2009 (Supplementary Table S1) [6, 7]. For NB without IDRFs, laparoscopic surgery could be performed safely because the tumor has no invasion of the important blood vessels or organs [8]. Therefore, IDRFs may play a more important role in affecting the performance of laparoscopic surgery than tumor size. In this study, we aimed to explore the criteria based on the IDRFs for laparoscopic surgery for NB and examine their safety and efficacy by comparing the clinical characteristics, perioperative indicators, postoperative complications, and prognosis with open surgery.

## Patients and methods

### Patients

We reviewed all pediatric patients with NB who were treated at Beijing Children’s Hospital (BCH) between December 2016 and January 2021, and 87 patients with NB without IDRFs were included in this study. The children were divided into open surgery and laparoscopic surgery groups. The data obtained included sex, age at diagnosis, clinical manifestation, tumor location, tumor size, INRG, treatment strategy, operating time, intraoperative bleeding, and the time to start postoperative feeding. Tumor size was evaluated based on the largest diameter assessed on the latest preoperative computed tomography. The study was approved by the Medical Ethics Committee of Beijing Children’s Hospital (2017-k-89).

### Treatment strategies

#### Chemotherapy

In this study, chemotherapy was administered according to the guidelines for the diagnosis and treatment of NB (2019) published by the National Health Commission of the People’s Republic of China [9]. Alternating chemotherapy regimens of CBVP (carboplatin, etoposide) were applied to children in the low- and intermediate-risk groups. Alternating chemotherapy regimens of CAV (vincristine, adriamycin, cyclophosphamide) and CVP (cisplatin, etoposide) were applied to children in the high-risk group.

#### Surgical procedure

**Fig. 1:**
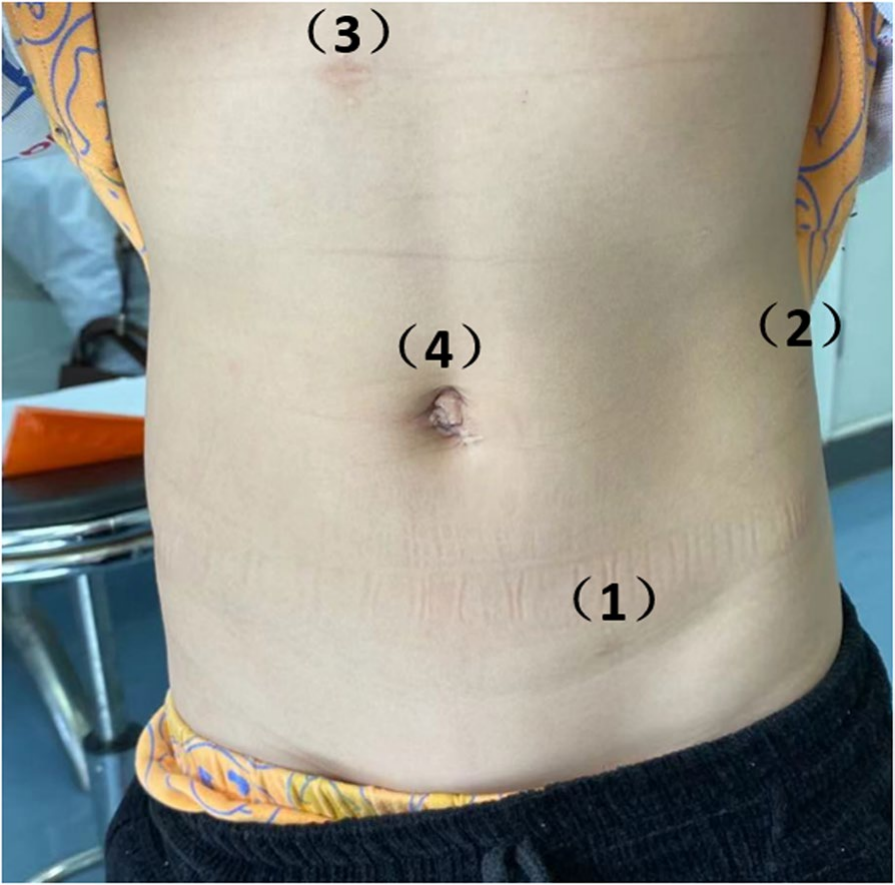
Site of ports for the left retroperitoneal tumor surgery. **1** Left midclavicular line extension on the hypogastrium; **2** intersection of the left anterior axillary line extension and the anterior superior spine; **3** subxiphoid; **4** incision of the umbilicus

**Fig. 2:**
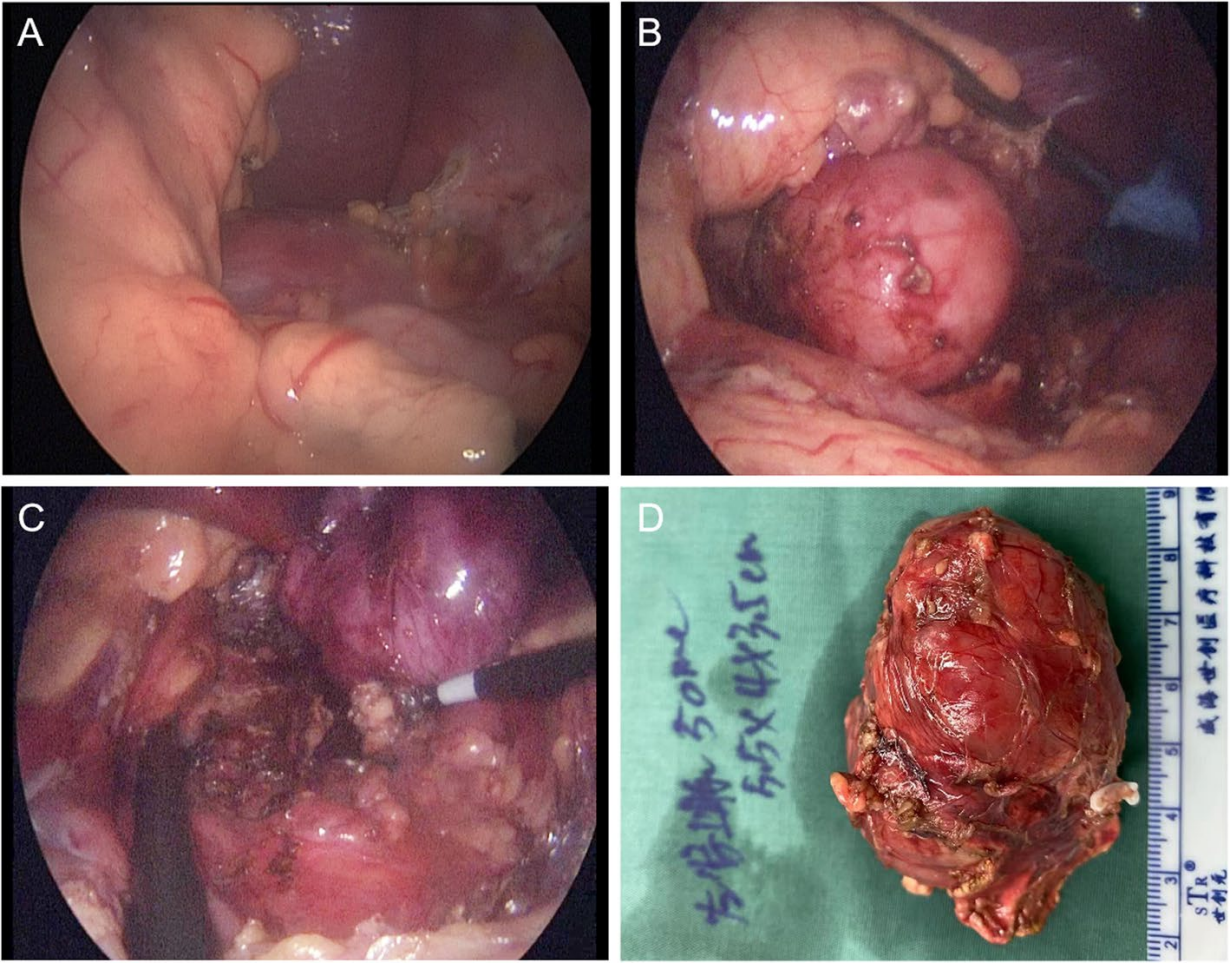
View of laparoscopic adrenal tumor resection. **A**–**C** View of laparoscopic adrenal tumor resection. **D** Surgical specimen

Patients with low- or intermediate-risk NB underwent resection of the primary tumor at the time of diagnosis or after three to four courses of chemotherapy. The patient was placed in a supine position, and the lower limbs were separated. The side with the tumor was padded at the level of the upper pole of the kidney. We elevated the side with the tumor to the 30° level by rotating the surgical bed. A 10-mm trocar was inserted through a 10-mm incision of the umbilicus using an electrotome to open the muscle layer. The abdominal cavity was insufflated with carbon dioxide to a maximum pressure of 10–12 mmHg, and gas flow was controlled at 0.5 L/min. Three additional 5-mm ports were inserted at these positions (Figs. 1 and 2): (1) midclavicular line extension of the tumor side on the hypogastrium; (2) intersection of the anterior axillary line extension on the tumor side and the anterior superior spine; (3) subxiphoid. The peritoneum around the adrenal gland was incised to expose the adrenal area, observe the tumor, and gradually separate the tumor from the surrounding tissue. Smaller vessels are usually coagulated by hook electrodes or ultrasonic scalpels, and the central vein of the adrenal gland is always clamped by a hemlock. The tumor was removed in an endoscopic bag through the extended umbilical port or the Pfannenstiel incision. Depending on the surgery, a drainage tube can be placed through the original incision for trocar.

### Statistical analysis

SPSS 20.0 statistical software was used to analyze and process the data. Continuous variables were tested for normality. Continuous variables with normal distributions are expressed as the mean ± standard deviation, and two independent sample T tests were used for intergroup comparisons. Continuous variables with a nonnormal distribution are expressed as the median (lower quartile, upper quartile), and the rank sum test was used for intergroup comparisons. The classified variables were represented by the number (percentage), and the intergroup comparison was performed by the *χ*^2^ test. Survival analysis was performed using the Kaplan‒Meier method, and the log rank method was used to test for significant differences. Data were considered statistically significant at *p* < 0.05.

## Results

### General information

From December 2016 to January 2021, 87 NB patients without IDRFs received surgery in our department. There were 41 (47.13%) females and 46 (52.87%) males, and the median age at diagnosis was 32 (10, 76) months. For the primary tumor site, 67 (77.01%) cases were from the adrenal, 15 (17.24%) cases were from the retroperitoneum, and 5 (5.75%) cases were from the pelvis. The largest tumor was 13.6 cm in diameter, and the median dimension of the tumor was 4.84 (± 2.69) cm. Based on the INRG staging system, 78 (89.66%) cases were classified as stage L1, 5 (5.75%) cases as stage M and 4 (4.59%) cases as stage MS. Regarding the status of *MYCN*, 7 cases were amplified, 70 were not amplified, and 10 were unknown. According to the INRG risk group, 76 (87.35%) cases were very low risk, 3 (3.45%) cases were intermediate risk and 8 (9.20%) cases were high risk. According to the International Neuroblastoma Pathology Classification (Shimada System), 62 (71.26%) cases were classified as favorable histology, and 25 (28.74%) cases were classified as unfavorable histology. The comparison of clinical characteristics between the laparoscopic and open surgery groups showed that there were no significant differences between the two groups in terms of the International Neuroblastoma Risk Group, the International Neuroblastoma Pathology Classification, etc., which indicated that the prognoses of the two groups were comparable (Table 1).

**Table 1:** Comparison of clinical features between laparoscopic and open surgery groups

Clinical features		Laparoscopic surgery (*n* = 33)	Open surgery (*n* = 54)	*p*
Gender (*n*, %)	Female	15 (45.45)	28 (51.85)	0.828
	Male	18 (54.55)	26 (48.15)	
Onset age (months)		22 (5, 44)	39 (21, 84)	0.008
Primary site (*n*, %)	Adrenal	32 (96.97)	35 (64.81)	0.001
	Retroperitoneal	1 (3.03)	14 (25.93)	
	Pelvic	0 (0.00)	5 (9.26)	
Maximum diameter of primary tumor (cm)		3.06 (1.37)	5.55 (2.77)	0.009
NSE (ng/mL)		22.80 (19.70, 34.60)	21.70 (19.00, 29.00)	0.636
LDH (U/L)		260.00 (226.00, 303.00)	244.00 (216.50, 295.50)	0.764
INPC (*n*, %)	Favorable	24 (72.72)	38 (70.37)	0.813
	Unfavorable	9 (27.20)	16 (29.63)	
*MYCN* status (*n*, %)	Not amplified	30 (90.90)	40 (74.07)	0.129
	Amplified	2 (6.06)	5 (9.26)	
	Unknown	1 (3.04)	9 (16.67)	
1p (*n*, %)	Normal	25 (75.75)	39 (72.22)	0.933
	Aberration	2 (6.06)	5 (9.26)	
	Unknown	6 (18.19)	10 (18.52)	
11q (*n*, %)	Normal	24 (72.72)	40 (74.07)	0.962
	Aberration	3 (9.09)	4 (7.41)	
	Unknown	6 (18.19)	10 (18.52)	
INRG stage (*n*, %)	L1	24 (72.73)	54 (100)	< 0.001
	M	4 (12.12)	0	
	MS	5 (15.15)	0	
INRG risk (*n*, %)	Very low	27 (81.82)	49 (90.74)	0.098
	Intermediate	3 (9.09)	0	
	High	3 (9.09)	5 (9.26)	

*NSE* neuron-specific enolase, *LDH* lactate dehydrogenase, *NB* neuroblastoma, *GNB* ganglioneuroblastoma, *GN* ganglioneuroma, *INPC* International Neuroblastoma Pathology Classification System, *INRG* International Neuroblastoma Risk Group

1. Continuous variables are presented as the median and interquartile range

2. Normally distributed variables are presented as mean and standard deviation

3. Classification variables are presented as numbers (percent)

4. Reference ranges of tumor markers: serum NSE ≤ 25 ng/mL; serum LDH 110 U/L-295 U/L

### Treatment

In the 33 patients in the laparoscopic surgery group, according to the INRG risk group, 27 patients were in the very low-risk group, 3 were in the intermediate-risk group and 3 were in the high-risk group. Three cases were treated with preoperative chemotherapy, and 14 cases were treated with postoperative chemotherapy. In the open surgery group, 8 patients received preoperative chemotherapy, and 27 received postoperative chemotherapy.

### Peri- and postoperative situations

In the laparoscopic group, three (3/33, 9.09%) cases were converted to open surgery due to the invasion of renal vessels. Regarding perioperative-related indicators (Table 2), there were statistically significant differences between the laparoscopic and open groups in terms of intraoperative bleeding (*p* = 0.013), time to start postoperative feeding (*p* = 0.002) and hospitalization expenses (*p* = 0.042); however, there were no significant differences in terms of operative time, intraoperative blood transfusion, hospital stay or postoperative complications (all *p* > 0.05). Specifically, the loss of blood volume during surgery in the laparoscopic group was 6.06 (2.00, 10.00) ml, and the mean time to start postoperative feeding was 2.83 (2.00, 3.00) days, which indicated that regarding intraoperative bleeding and postoperative recovery, the laparoscopic group was superior to the open group. In terms of postoperative complications, this study counted postoperative indicators of abnormal adrenocortical function, hemorrhage, intestinal obstruction, hypertension [10], celiac leakage [11], pleural effusion, diarrhea, wound dehiscence, and neurological dysfunction. Postoperative complications were reported by Clavien-Dindo Classification (CDC) system (Supplementary Table S2) [12] and comprehensive complication index (CCI) [13]. In this study, five cases had postoperative complications (Table 3), 3 cases were in the open surgery group, 1 case had frequent diarrhea (noninfectious) > 7 days, and 2 cases had adrenocortical insufficiency. Two cases were in the laparoscopic surgery group: 1 case had adrenocortical insufficiency, and the other had pleural effusion. After conservative treatment, all the patients recovered from the complications.

**Table 2:** Comparison of peri- and post-operative variables between laparoscopic surgery and open surgery groups

	Laparoscopic surgery (*n* = 33)	Open surgery (*n* = 54)	*p*
Operative time (min)	194.97 (151.00, 226.50)	190.06 (139.75, 232.75)	0.740
Loss of blood volume (ml)	6.06 (2.00, 10.00)	8.36 (5.00, 10.00)	0.013
Intraoperative blood transfusion (*n*, %)	0	0	–
Time to start postoperative feeding (days)	2.83 (2.00, 3.00)	3.49 (3.00, 4.00)	0.002
Hospital stay (days)	9.54 (6.00, 10.50)	9.85 (8.00, 11.00)	0.231
Postoperative complications (*n*, %)	2 (6.06)	3 (5.56)	0.632
hospitalization expenses (¥)	38,400.06 (32,674.47, 44,788.74)	35,721.25 (27,855.27, 41,095.50)	0.042
Recurrence (*n*, %)	0	0	–
Follow-up period (months)	26.00 (16.00, 44.00)	32.00 (29.00, 41.00)	0.051

1. Continuous variables are presented as the median and interquartile range

2. Normally distributed variables are presented as mean and standard deviation

3. Classification variables are presented as numbers (percent)

**Table 3:** Postoperative complications in the children with NB

Patient	Group	Complication	CDC	CCI	Treatment	Outcome
Case 1	Open surgery	Diarrhea^a^	Grade II	20.9	Rehydration and symptomatic treatment	Cure
Case 2	Open surgery	Adrenocortical^a^ insufficiency	Grade II	20.9	Hydrocortisone	Cure
Case 3	Open surgery	Adrenocortical insufficiency	Grade II	20.9	Hydrocortisone	Cure
Case 4	Laparoscopic surgery	Adrenocortical insufficiency	Grade II	20.9	Hydrocortisone	Cure
Case 5	Laparoscopic surgery	Pleural effusion^a^	Grade III a	26.2	Retained chest drainage tube	Cure

CCI calculator is available at website (http://www.assessurgery.com)

*CDC* Clavien-Dindo Classification, *CCI* comprehensive complication index (CCI)

^a^Diarrhea: three or more abnormally loose or watery stools a day, more than 1 week in duration, laboratory tests suggest non-infectious. Adrenocortical insufficiency: on the first postoperative day, serum cortisol concentration was less than 5 μg/dl with disturbed water-electrolyte balance. Pleural effusion: pathologic accumulation of fluid in the pleural space

### Prognosis

All patients were followed up until February 2022 in this study, with a median follow-up time of 31 (25, 41) months. Eighty-six patients survived without disease, and 1 patient in the open surgery group was lost to follow-up. There was no recurrence or progression in either group during the follow-up, and the 3-year overall survival rate and event-free survival rate were 100%.

## Discussion

NB is the most common extracranial solid tumor in children, and surgery is the mainstay of treatment for localized NB. Because of its deep anatomical location and difficulty in exposure, NB has long been operated mainly by open surgery. However, with the development of laparoscopic technology, it has also been used more often in selective NB tumors. In some studies, laparoscopic surgery for smaller benign adrenal tumors (< 6 cm in diameter) has been shown to be safe and effective in terms of perioperative and long-term follow-up [14, 15]. Marc-David Leclair et al. also showed that good local control can be obtained with laparoscopic treatment for some children with low- and intermediate-risk NB [16]. In this study, we found that in terms of prognosis and surgical complications, there was no significant difference between the laparoscopic and open surgery groups, supporting the safety of laparoscopic surgery.

Regarding the indications for the application of laparoscopic surgery for NB, most studies have focused on tumor size and vascular encasement. It has been suggested that laparoscopic resection is safe and effective for NB with a tumor diameter < 2 cm [17]. P. De Lagausie et al. showed that adrenal NB with a diameter less than 6 cm and no adjacent vascular or organ involvement could be completely removed by laparoscopic surgery [18]. Takafumi Kawano indicated that the maximum diameter of the NB that can be resected by laparoscopy should be judged according to the height of the child, and almost all of the tumors that can be safely resected are under 5–8 cm in diameter [19]. However, Ezekian et al. reported that a large proportion of children with NB > 6 cm in diameter can be safely and effectively treated with laparoscopy, but this may require the tumor to have no IDRFs, and the surgeon has extensive experience in the procedure [20]. In this study, children in the laparoscopic group had smaller tumor diameters than those in the open group, but two children had tumors > 5 cm in diameter, 5.5 cm and 8.7 cm. In terms of the choice of laparoscopic surgery, this study suggests that the absence of IDRFs may be a more important factor than tumor size. If the localized tumor was confined to one body compartment and did not involve the list of IDRFs, laparoscopic surgery for tumor removal could be possible. Of course, this conclusion still needs to be verified in large prospective clinical trials.

The application of laparoscopy for the treatment of malignant solid tumors may have the possibility of incisional implantation or metastasis, which is more frequently reported in adults [21, 22]. Incisional implantation and metastasis may be associated with tumor cell shedding during tumor resection [23]. To prevent incisional implantation and metastasis, the tumor should be removed using an endoscopic retriever to prevent contact between the tumor and the incision. If the tumor is large and difficult to remove through the original umbilical incision, an enlarged umbilical incision or Pfannenstiel incision may be used. The tumor should be removed as completely as possible to avoid being smashed to pieces in the endoscopic retriever. The lower abdominal fold provides an ideal site for the extraction of tumors with diameters greater than 5 cm. The children in this study had a tumor diameter of 8.7 cm, and the tumor was removed completely through the Pfannenstiel incision. Compared to conventional surgical incisions, it has a better cosmetic appearance, although the incision was also enlarged.

In general, laparoscopic surgery is characterized by a short operative time, fast recovery, low incidence of bowel adhesion formation decreases, and better cosmetic appearance [3, 8, 24]. Although there were no statistically significant differences in operative time or postoperative complications between laparoscopic and open surgery in our study, laparoscopic surgery still had some advantages in terms of intraoperative bleeding and time to start postoperative feeding. In terms of operation time, it was similar between laparoscopic surgery and open surgery group (194.97 min versus 190.06 min). The operation time of laparoscopic surgery group prolonged because of four cases with stage M and five cases with stage MS were included in this study. Among these 9 cases with metastasis, in addition to resection of the primary tumor, liver biopsy was performed in three (3/9) cases, and subcutaneous mass or lymph node biopsy was performed in six (6/9) cases. Because laparoscopic surgery has a smaller incision and clearer view, a more detailed and safer tumor resection could be achieved, and intraoperative bleeding was significantly lower than in the open group (*p* = 0.013). Children in the laparoscopic surgery group started eating earlier than the children in the open surgery group (*p* = 0.002). This will allow children with NB to receive faster nutritional support and earlier postoperative chemotherapy. Laparoscopic surgery also allows for maximum organ preservation. In the laparoscopic group of this study, there were two cases with bilateral NB; in one case, both adrenal glands were preserved, and in the other case, due to the large tumor on the right side, approximately 3.5 cm in diameter, there was no obvious residual adrenal gland, and only part of the left adrenal gland was preserved.

Regarding prognosis, there were no significant differences between the open and laparoscopic groups in this study. All 33 patients in the laparoscopic group underwent complete resection of the primary tumor. The 3-year EFS and OS of these children were both 100%, which may be related to the high proportion of very low-risk children (27/33) and the short follow-up period. On the other hand, it shows that laparoscopic surgery for NB with no IDRFs is effective and does not raise the risk of local recurrence of the tumor. Similar results have been reported by Marc-David Leclair et al. [16] in a multicenter retrospective study including 45 children with NB treated with laparoscopic surgery and Zenitani M et al. [8] in a national survey of laparoscopic resections for pediatric malignancies among 72 cases of localized NB.

For children with stage MS NB, laparoscopic surgery allows not only resection of the primary tumor but also liver biopsy. Three (3/9) children with MS stage underwent laparoscopic liver biopsy in this study. The choice of position and incision for patients requiring liver biopsy differs from that for simple resection of adrenal tumors. For resection of the adrenal tumor alone, we can choose the lateral position. If liver biopsy is desired, the supine position is a better choice, and the surgery requires additional incisions. Laparoscopic adrenal tumor resection via the retroperitoneal approach is technically mature and has been reported in related cases [16, 25], but the retroperitoneal approach does not allow adequate examination of the child’s abdominal cavity and does not allow for related procedures such as liver biopsy. All children in this study had a transperitoneal approach.

However, this study was a single-center retrospective study with no randomization in the choice of laparoscopic treatment. In the past, our decision to perform laparoscopic surgery was mainly based on the IDRFs, tumor size, and experience of the surgeon, there existed selection bias. The follow-up of this study was also not long enough. The indication of choosing laparoscopic surgery for NB and the impact of laparoscopic resection on NB postoperative metastasis, recurrence and long-term survival still need to be validated in prospective clinical trials with large samples.

## Conclusion

For children with localized NB who have no IDRFs, laparoscopic surgery can be performed safely and effectively. Tumor size may not be a significant factor limiting the use of laparoscopy. It has equal recurrence and survival rates compared with open resection and provides some advantages of minimally invasive surgery.

## Supplementary Information

**Additional file 1: Supplementary Table 1.** Descriptions of Image-Defined Risk Factors.

**Additional file 2: Supplementary Table 2.** Classification of Surgical Complication.

## Data Availability

All data generated or analyzed during this study are included in this published article and its supplementary information files.
