# Supplementary material for: Safety and feasibility of laparoscopic resection of abdominal neuroblastoma without image-defined risk factors: a single-center experience
Source: World J Surg Oncol. 2023 Mar 28;21:113. doi: 10.1186/s12957-023-02997-9 (PMC10044736; doi:10.1186/s12957-023-02997-9)
Supplement: Supplementary file 1 — Additional file 1: Supplementary Table 1. Descriptions of Image-Defined Risk Factors. [file 12957_2023_2997_MOESM1_ESM.docx]

| **Supplementary Table 1.** Descriptions of Image-Defined Risk Factors |
| --- |
| **Ipsilateral tumor extension within two body compartments** |
| Neck-chest, chest-abdomen, abdomen-pelvis |
| **Neck** |
| Tumor encasing carotid and/or vertebral artery and/or internal jugular vein |
| Tumor extending to base of skull |
| Tumor compressing the trachea |
| **Cervico-thoracic junction** |
| Tumor encasing brachial plexus roots |
| Tumor encasing subclavian vessels and/or vertebral and/or carotid artery |
| **Tumor compressing the trachea** |
| Thorax |
| Tumor encasing the aorta and/or major branches |
| Tumor compressing the trachea and/or principal bronchi |
| Lower mediastinal tumor, infiltrating the costo-vertebral junction between T9 and T12 |
| **Thoraco-abdominal** |
| Tumor encasing the aorta and/or vena cava |
| **Abdomen/pelvis** |
| Tumor infiltrating the porta hepatis and/or the hepatoduodenal ligament |
| Tumor encasing branches of the superior mesenteric artery at the mesenteric root |
| Tumor encasing the origin of the coeliac axis, and/or of the superior mesenteric artery |
| Tumor invading one or both renal pedicles |
| Tumor encasing the aorta and/or vena cava |
| Tumor encasing the iliac vessels |
| **Pelvic tumor crossing the sciatic notch** |
| **Intraspinal tumor extension whatever the location provided that:** |
| More than one third of the spinal canal in the axial plane is invaded and/or the perimedullary leptomeningeal spaces are not visible and/or the spinal cord signal is abnormal |
| **Infiltration of adjacent organs/structures** |
| Pericardium, diaphragm, kidney, liver, duodeno-pancreatic block, and mesentery |
| **Conditions to be recorded, but not considered IDRFs** |
| Multifocal primary tumors |
| Pleural effusion, with or without malignant cells |
| Ascites, with or without malignant cells |
| **Abbreviation:** IDRFs, image-defined risk factors. |
| **Source：**Reference 6. The International Neuroblastoma Risk Group (INRG) classification system: an INRG Task Force report. |
