# Supplementary material for: Safety and feasibility of laparoscopic resection of abdominal neuroblastoma without image-defined risk factors: a single-center experience
Source: World J Surg Oncol. 2023 Mar 28;21:113. doi: 10.1186/s12957-023-02997-9 (PMC10044736; doi:10.1186/s12957-023-02997-9)
Supplement: Supplementary file 2 — Additional file 2: Supplementary Table 2. Classification of Surgical Complication. [file 12957_2023_2997_MOESM2_ESM.docx]

| **Supplementary Table 2.** Classification of Surgical Complication | |
| --- | --- |
| **Grade** | **Definition** |
| Grade I | Any deviation from the normal postoperative course without the need for pharmacological treatment or surgical, endoscopic, and radiological interventions  Allowed therapeutic regimens are: drugs as antiemetics, antipyretics, analgetics, diuretics, electrolytes, and physiotherapy. This grade also includes wound infections opened at the bedside |
| Grade II | Requiring pharmacological treatment with drugs other than such allowed for grade I complications  Blood transfusions and total parenteral nutrition are also included |
| Grade III | Requiring surgical, endoscopic or radiological intervention |
| Grade IIIa | Intervention not under general anesthesia |
| Grade IIIb | Intervention under general anesthesia |
| Grade IV | Life-threatening complication (including CNS complications)* requiring IC/ICU management |
| Grade IVa | Single organ dysfunction (including dialysis) |
| Grade IVb | Multiorgan dysfunction |
| Grade V | Death of a patient |
| Suffix “d” | If the patient suffers from a complication at the time of discharge (see examples in Table 2), the suffix “d”(for “disability”) is added to the respective grade of complication. This label indicates the need for a follow-up to fully evaluate the complication |
| *Brain hemorrhage, ischemic stroke, subarrachnoidal bleeding, but excluding transient ischemic attacks. CNS, central nervous system; IC, intermediate care; ICU, intensive care unit **Source:** Reference 10. Classification of surgical complications: a new proposal with evaluation in a cohort of 6336 patients and results of a survey. | |
